# Mycobacterium Chimaera: A Rare Presentation

**DOI:** 10.7759/cureus.2750

**Published:** 2018-06-06

**Authors:** Jeffrey A Miskoff, Moiuz Chaudhri

**Affiliations:** 1 Medicine, Jersey Shore University Medical Center, Neptune City, USA

**Keywords:** case report, coronary bypass surgery, mycobacterium, mycobacterium avium complex-mac, heater-cooler, open heart procedures, dna polymerase chain reaction, opportunistic infections, primary care

## Abstract

Mycobacterium chimaera is an indolent nontuberculous mycobacterium which is abundant in soil, dust, and water. Although lacking recognition garnered by other mycobacteria (i.e., M. tuberculosis), it has been recognized as an emerging opportunistic threat to patients undergoing coronary bypass surgery and open heart procedures requiring extracorporeal devices. Here, we present a case of an individual initially seen in the inpatient setting without a history of such procedures or other risk factors commonly associated with mycobacterium-laden infections.

## Introduction

Mycobacterium chimaera is a slow-growing nontuberculous mycobacterium grouped with Mycobacterium intracellulare and Mycobacterium avium to make up the Mycobacterium avium complex. Evidence suggests that it is responsible for symptoms similar to those found in disseminated mycobacterial diseases, prosthetic valve endocarditis, ocular emboli, vertebral osteomyelitis, hepatitis, and renal dysfunction, along with other life-threatening conditions [1-3]. Additionally, it can cause pneumonia in patients with underlying respiratory complaints, such as cystic fibrosis and other immunodeficiencies [4].

## Case presentation

A 67-year-old male presented to our care on December 26, 2017, for shortness of breath on exertion and persistent cough for three weeks. Prior to these symptoms, he was completely asymptomatic. Symptoms progressed gradually up to the extent that he was not able to leave his house. Furthermore, the patient experienced severe shortness of breath when he attempted to use the restroom. He described a productive cough with white to yellow sputum. Additionally, he felt feverish on three different occasions since the onset of symptoms. Although he had no chest pain, the patient experienced chest tightness and heaviness. The patient reported orthopnea and waking up in the middle of the night due to shortness of breath. The patient denied palpitations, chills, night sweats, dizziness, recent travel, contact with birds, exposure to tuberculosis, and contact with wild animals. On presentation, his blood pressure ranged from 95/57 to 125/63 mmHg, a heart rate of 98 beats per min, a respiration rate of 19 breaths per min, and temperature of 97.3 F. His oxygen saturation was 94% on room air. Pulmonary function testing revealed a Gold 3 category with a forced expiratory volume in 1 second (FEV-1) of 41%, forced vital capacity (FVC) of 69%, and an FEV-1/FVC ratio of 47%. Lastly, the patient's bicarbonate, electrolytes, platelets, and transaminases were within normal limits. Physical examination findings of a barrel-shaped chest and poor air exchange, combined with the results of pulmonary function testing, supported a diagnosis of chronic obstructive pulmonary disease (COPD).             

**Figure 1:**
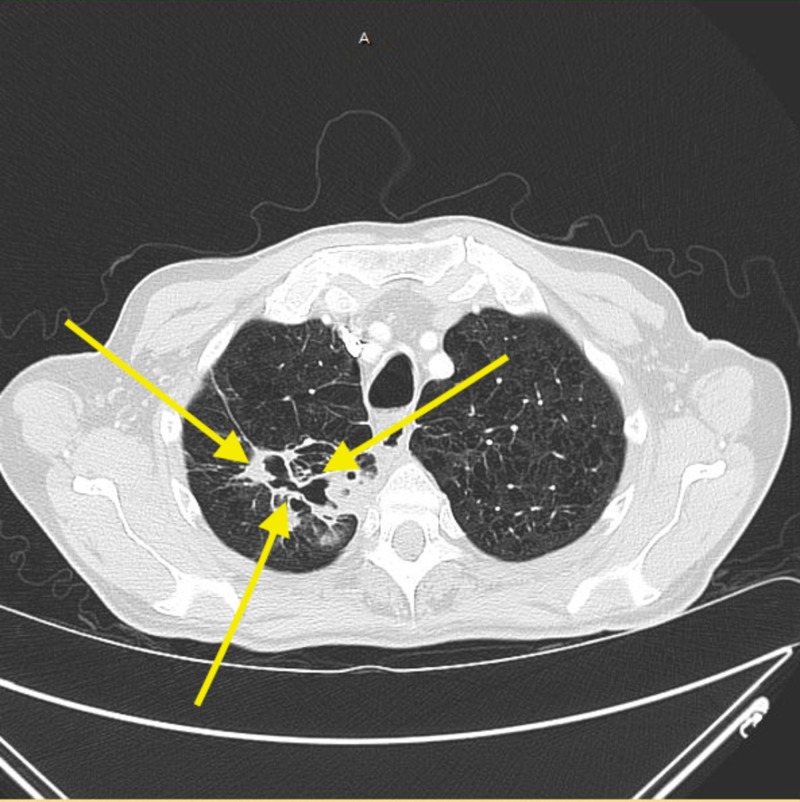
Computed tomographic angiography ( CTA of the patient's chest illustrating cavitation extending up to the pleural surface, along with patchy reticulonodular infiltrates (yellow arrows).

In addition, a chest x-ray exhibited right-sided apical opacity. The patient underwent computed tomography angiography (CTA), which was negative for pulmonary embolism and illustrated moderate to marked opacity in the right lung apex with cavitation extending up to the pleural surface. Additionally, patchy reticulonodular opacities consistent with infiltrates were present in the right upper, right middle, right lower, and left upper lobe (Figure 1). These findings led to a diagnosis of cavitary pneumonia, and blood cultures, along with mycoplasma IgM, were ordered. In addition, the patient tested negative for human immunodeficiency virus (HIV-1 and 2). The infectious disease team was consulted for their expertise and to ensure a thorough workup. Although the patient did not have true exposure to tuberculosis, the QuantiFERON®-TB Gold test (QFT-G) (Qiagen, Hilden, Germany) was ordered and the results for active and latent tuberculosis were negative. Furthermore, acid-fast bacilli (AFB) sputum cultures, fungal stains, serologies, and β-D-glucan were negative as well. Due to inconclusive results, a bronchoscopy with biopsies was performed which yielded a necrotic black specimen. A subsequent culture grew Mycobacterium chimaeraand confirmed the diagnosis.

Although a diagnosis was reached, this patient needed further workup to ensure complete resolution of this condition. The infectious disease team suggested optimizing the patient's respiratory regimen, following up with a chest CT in three months, and treating with a multidrug regimen for atypical mycobacterial infection if the patient clinically worsened.

## Discussion

Mycobacterium avium complex (gram-positive, nonmotile, and acid-fast) is a group of mycobacteria comprising of intracellulare, avium, and chimaera. Mycobacterium chimaera (M. chimaera) has been associated with low virulence, and until recently, it remained a mystery. Although M chimaera was successfully isolated from water samples in 2012, it was not linked with infections in patients undergoing cardiac surgical procedures until 2013 [4]. Unfortunately, this organism did not come to the forefront until 2015, when this infectious agent was identified as a culprit responsible for two prolonged outbreaks involving prosthetic valves and associated systemic infection in the United States and Europe [1, 3]. Specifically, investigations revealed that the contaminated heater-cooler devices (Stockert 3T) came from one particular manufacturing facility, LivaNova PLC, in London [5]. The importance of heating-cooling units lies in their ability to regulate the body temperature of the patients during cardiac surgery; evidence suggests that the airborne transmission of aerosolized bacteria from the water tanks was responsible for this infection [6]. Isolates harvested from the bone marrow of a patient who underwent cardiac surgery were identical to two isolates from a single heating and cooling unit used in the same facility when the patient was operated on again [5-6]. These findings provided further evidence that strengthened the association between the units and M. chimaera. The research found that M. chimaera was predominantly found in water supply installations and could be diagnosed using 16S ribosomal RNA (rRNA) gene sequencing [3].

In addition to utilizing unique ribosomal sequencing, this group needs to be incubated for at least seven days at a specific temperature of 37° C for successful isolation [3]. Literature provides sufficient evidence associating Mycobacterium chimaera as the infectious organism in patients undergoing cardiac surgery (specifically, surgeries utilizing heater-cooler units) [7]. Our patient did not undergo coronary bypass surgery nor other procedures requiring these units, thus making this a unique presentation. Moreover, evidence suggests that the infection becomes clinically apparent after a lag time of months to years and this plays a significant role in M. chimaera remaining undiagnosed [8]. In a different study, investigators collected water and air samples from the units, wards, water circuits of heater-cooler units, and the operating room and isolated M. chimaera by utilizing random amplified polymorphic deoxyribonucleic acid (DNA) polymerase chain reaction (RAPD-PCR). Moreover, they determined that patients undergoing open heart surgery showed no signs of infection until 1.5 to 3.6 years after the procedure [3, 7]. Furthermore, the infection remains clinically dormant for years, making a diagnosis of Mycobacterium chimaera in a timely manner challenging [2, 6].

## Conclusions

M. chimaera is a rare species of atypical nontuberculous mycobacterium typically found in association with cardiac surgery patients. The above patient did not have the typical risk factors for M. chimaera, which makes this case unique. The decision to treat the patient or not presents several challenges, especially in light of severe emphysema and structural lung disease on imaging. Stabilizing the patient’s COPD and concomitant consultation with an infectious disease specialist was the first step. Ultimately, the patient may require a prolonged multidrug regimen to eradicate the infection if there is any further clinical or radiographic deterioration. Treatment of M. chimaera* *is the same as other Mycobacterium avium complex infections, including two to three antimicrobials for at least 12 months and often up to 18 months. Commonly used first-line drugs include macrolides, ethambutol, and rifamycins. Aminoglycosides, such as streptomycin and amikacin, are also used as additional or alternative agents. Treatment regimens may differ, especially if sensitivity testing reveals a resistant organism. The patient may also express concerns about potential adverse drug reactions and refuse or not tolerate a prolonged treatment regimen.
